# Patients’ Satisfaction With Pharmaceutical Care Services Provided During COVID Pandemic: Experience From Greece

**DOI:** 10.7759/cureus.37743

**Published:** 2023-04-18

**Authors:** Konstantinos Georgiopoulos, Panagiota Manthou, Anna Korompeli, Panagiotis Vasileiou, Georgios Lioliousis, Dimitris Dainavas, Ioustini Pietri

**Affiliations:** 1 Intensive Care Unit, Thoracic Diseases General Hospital Sotiria, Athens, GRC; 2 Nursing, National and Kapodistrian University of Athens, Athens, GRC; 3 ICU GONK "Agioi Anargyroi" General Hospital, National and Kapodistrian University of Athens, Athens, GRC; 4 Laboratory of Histology & Embryology, National and Kapodistrian University of Athens, Athens, GRC; 5 Critical Care Unit, Thoracic Diseases General Hospital Sotiria, Athens, GRC; 6 Intensive Care Unit, 1st Department Respiratory Medicine, Thoracic Diseases General Hospital Sotiria, Athens, GRC; 7 ICU, General Oncology Hospital of Kifisia, Athens, GRC; 8 Nursing, General Oncology Hospital of Kifissia, Athens, GRC

**Keywords:** first aid, covid pandemic, customer satisfaction, pharmacy, primary health care

## Abstract

Introduction

Private pharmacies can contribute to the health care system through primary care.

Purpose

The purpose of this study is to determine patients' expectations of pharmaceutical care services during covid pandemic in order to measure the level of patient satisfaction provided by the Greek healthcare system. Also, it is important to identify the associated factors that might affect patient satisfaction.

Material and Method

The sample of the study consisted of 168 customers of pharmacies in Athens. A patient satisfaction survey was conducted at health facilities operating in Athens. Data regarding socio-demographic characteristics and parameters that measure patients' expectations and satisfaction were collected through a closed-ended questionnaire that had been tested for validity and reliability. The patient’s point of view was evaluated based on their expectation and perception of the pharmaceutical care services they had received. Data were entered into SPSS version 22 (IBM Corp, Armonk, NY ), and descriptive statistics, cross-tabs, and binary logistic regressions were utilized. P < 0.05 was used to declare association.

Results

About 89.3% of the participants were insured in the Greek health system. The main reason for visiting the pharmacy was the purchase of medicines and products (95.2%), vaccinations (19.6%), and consulting services for first aid (17.3%). The pharmacist was rated for his courtesy, willingness, friendliness, and reliability. Only 48.2% of participants knew that the pharmacy provided primary care services during the pandemic. The most common services provided were blood pressure measurement and intramuscular injections. Around 64.2% of them were fully satisfied. Pharmacists in primary care teams are uniquely positioned to facilitate practice expansion and make medicine a trusted resource for physicians, as well as improve health outcomes for patients.

Conclusions

The pharmacy has a leading role in health care due to easy access, and fast and immediate service. Patient-clients in Greek society trust their pharmacist as a health professional. Further research is suggested to ensure that through the delivery of health services by pharmacies, the cost of primary care could be lower.

## Introduction

The role of the pharmacist in the primary health center involves both direct care services (assessment for the correct use of medicines, education of patients, etc.) as well as services implemented by other members of the health care team of primary care (counseling services when a medicine is supplied repeatedly, dosage administration aids, health promotion strategies, research, etc.) [1]. Pharmacists provide direct patient care under the pharmaceutical patient care model. Pharmaceutical care aims to improve the patient's quality of life. Communication with patients, prioritization of needs, and an understanding of therapeutic goals are all part of this process [2-3]. Specialty pharmacists who focus on high-cost, high-touch medication therapy for patients with complex disease states, may have a broader scope of practice in Canada. Monitoring and adherence to treatment, assessment, prioritizing emergency or hospital transfers, home visits, and health promotion education can all be done with expert pharmacists. As a matter of course, guidelines have been provided to guide pharmacists in the process of including them in primary healthcare teams [4].

Considering the intense competition in pharmaceutical retail, pharmacies must ensure that customer-patient satisfaction remains a top priority. Pharmacies play a crucial role in providing medication, primary care, monitoring, and referring patients to other health professionals. The quality of healthcare services can be measured by patient satisfaction [4]. In the primary healthcare setting, a measure of patient satisfaction is an effective parameter for improving services. Tracking and identifying changes in customer requirements is part of determining patient satisfaction, especially during pandemics. Services can be improved based on the results [5, 6]. 

Private pharmacies in Greece are being forced to contribute more to the health system due to the COVID-19 epidemic. The study aimed to understand the role of pharmacists in Greece during the pandemic and the patient's expectations. The findings help to understand the importance of community pharmacists in an emergency situation.

## Materials and methods

A literature review was performed on many electronic databases to identify relevant studies that are focused on the role of pharmacists and patients' satisfaction in the COVID-19 pandemic. The search was limited to English-published articles focused on the topic.

To collect research data, an anonymous questionnaire was developed for the purposes of this study. The multiple-choice questions were graded on a Likert scale. The first part of the survey asked about demographic data such as gender and age, and the second part asked about the services provided and patient satisfaction. The patients in this research were self-medicated at pharmacies during the COVID-19 epidemic.

Inclusion and exclusion criteria

To reduce bias, sample criteria had to be determined. Inclusion criteria were patients between the ages of 18 and 70; patients who were capable of reading and writing; patients who signed informed consent; and patients who visited Athens pharmacies and received pharmaceutical services. Those who failed to complete the questionnaire completely and those with reading difficulties without assistive devices were excluded from the study. The validity and reliability of the questionnaire instrument were tested using SPSS (IBM Corp. Released 2013. IBM SPSS Statistics for Windows, Version 22.0. Armonk, NY: IBM Corp).

A total of 168 patients were enrolled in the study. A mean value (mean), standard deviation (SD), median (median), and interquartile range (IQR) were used to describe quantitative variables. Absolute (N) and relative (%) frequencies were used to describe the qualitative variables. Pearson's x2 test or Fisher's test was used to compare the proportions exact test where necessary. To compare quantitative variables between two groups, the non-parametric Mann-Whitney test was used. To find independent factors related to knowledge and use of services of primary health care at the pharmacy, a logarithmic regression analysis was performed (logistic regression analysis) with the sequential inclusion/removal process (stepwise) and relative ratios (odds ratio) with their 95% confidence intervals (CI) were obtained. Significance levels are two-sided and statistical significance was set at 0.05.

## Results

Sociodemographic characteristic

Among the respondents, it was found that 44.0% of respondents aged were over 40 years and 69.6% of participants were female. About half of the participants (52.4%) were married. According to the study findings, the majority of the samples with the most recent education level of respondents include senior high school (39 respondents), and higher education (university level) (117 respondents). This is in accordance with World Self Medication Industry research, which found that self-medication behavior increased in the population with higher education levels both before and after the COVID-19 pandemic [7] (Table 1).

**Table 1:** Sociodemographic character of respondents.

		Ν	%
Gender	Female	117	69,6
	Male	51	30,4
Age	< 25	25	14,9
	25-30	16	9,5
	31-35	25	14,9
	36-40	28	16,7
	>40	74	44,0
Family Condition	not married	60	35,7
	married	88	52,4
	divorced	15	8,9
	widow	5	3,0
Education	Elementary School	9	5,4
	High School	39	23,2
	Bachelor Degree	71	42,3
	Master Degree	31	18,5
	Phd Degree	15	8,9
	Other	3	1,8
If other, specify	Private college	1	0,6
	Vocational Training Institute	2	1,2
Work	Public Sector	55	32,7
	Private Sector	50	29,8
	Student	17	10,1
	Entrepreneur	21	12,5
	Unemployer	11	6,5
	Retired	14	8,3
Health Insurance	No	18	10,7
	Yes	150	89,3

About 34.5% of participants visited the pharmacy once a month, and 24.4% two to three times a month. Additionally, 53.0% of participants visited a pharmacy nearby and 33.3% of the participants have visited a specific nearby pharmacy for at least five years. The primary reason for visiting a pharmacy was to purchase drugs and medical products. Other reasons included vaccinations, drug counseling, rapid tests for COVID-19 or influenza, etc. It was reported that almost all participants (99%) were satisfied with both available pharmaceuticals and non-pharmaceutical products, especially at the e-shop (Table 2).

**Table 2:** Reasons of visiting the pharmacy

		Ν	%
Frequency	2-3 times per week	27	16,1
	Once a week	28	16,7
	2-3 times per month	41	24,4
	Once a month	58	34,5
	Rarely	14	8,3
Reason of visitting*	Drugs and other medical products	160	95,2
	Immediate anti pregnant therapy	7	4,2
	Check arterial blood pressure	13	7,7
	Check glucose	4	2,4
	Check weight	22	13,1
	Vaccinations	33	19,6
	Psychological support	11	6,5
	Rapid Test for Covid/ Influensa	29	17,3
	Drug Recycle	25	14,9
	First Aid	8	4,8
	Cosmetic	1	0,6
	Pediatric Products	1	0,6
	Pregnant Counseling	1	0,6
	Counseling	3	1,8
Drug/Product Sufficient Supply;	No	2	1,0
	Yes	166	99,0

The participants rated their pharmacists highly for politeness, willingness, friendliness, and reliability. As well as their competence and secrecy, they received quite high ratings. Among participants, 48.2% knew if the pharmacy they visit most often provides primary care services. Blood pressure measurements and intramuscular injections were the most frequent services pharmacies provided. Ninety percent of participants who knew their most frequent pharmacy provided primary care services, used them to access these services. Sixty-four percent were completely satisfied, and 86.4% chose it because they trusted the pharmacist, while 28.4% chose it because it was easy to access (Table 3).

**Table 3:** Primary care services from pharmacies

		Ν	%
Do you know if your preferred pharmacy offers primary care services?	No	87	51,8
	Yes	81	48,2
If yes, specify?	First Aid	6	7.4
	Wound Change	3	3,7
	Vaccinations	6	7,4
	IM	25	30,9
	Arterial Blood Pressure	30	37,0
	Glucose Check	3	3,7
	Rapid Tests	5	6,2
	Other	3	3,7
Have you ever used primary care services?	No	8	9,9
	Yes	73	90,1
Reason for primary care services at pharmacy?	Trust	70	86,4
	Send by other health care specialist	2	2,5
	cost	9	11,1
	Quick service	14	17,3
	Easy access	23	28,4
	other	1	1,2

Participants who were trained by their pharmacists on health education issues such as smoking cessation, cardiovascular disease prevention, etc. used primary care pharmacy services significantly more than participants who weren't trained. When asked about the pharmacist’s role, most respondents had a good understanding of the steps involved in checking medication appropriateness as part of the dispensing process (Table 2). However, many respondents agreed that the pharmacist and not the family doctor tells them what to do when they miss a dose of their medication (63%), or advice them about the covid measures if someone is positive (40%).

Patients indicated a high level of trust that the pharmacist would keep their health information confidential (65%). Over half of the respondents (52%) agreed the pharmacist was the best person to help them manage the side effects of a medication or encourage them to ask for a second opinion from the family doctor.

A multivariate logarithmic regression analysis was conducted using the pharmacy's primary care services as the dependent variable and the demographics and other data of the participants as independent variables. The probability of using primary care services from a pharmacy was 2.33 times higher than for participants who were not trained in such matters (95% CI: 1.78 - 3.06, p = 0.002).

## Discussion

The purpose of this study was to assess the knowledge and satisfaction of pharmacy customers who visit the pharmacies of Athens regarding the primary care services provided by the pharmacies, as well as their reasons for being satisfied with the pharmacist.

Studies have shown that patients are more satisfied with primary care services if they are associated with interpersonal communication [8]. According to a study conducted in two centers in Egypt on patient satisfaction with primary health care services, 52.2% and 81.2% of participants were satisfied with the pharmacy's services [9]. 

According to another study on the correlation of patient satisfaction with physician services in primary care centers, high levels of client satisfaction were positively correlated with good physician relationships. This enhanced patient compliance [10]. According to our research, participants were very satisfied with the information provided. Patients reported similar findings regarding the provision of counseling services. In primary care services in Sweden, it was found that the provision of information by pharmacy staff was rated higher by patients than that received by the medical community in terms of consultation and interpretation when analyzing factors contributing to patient satisfaction [11].

Klepser et al. proposed a pharmacy implementation protocol to reduce the complications and costs associated with seasonal influenza and pharyngitis disease. To provide initial training for pharmacists, the researchers used a certificate program and shared standards from previously validated models. Pharmacy companies were responsible for navigating all applications within their organization. In total, 661 visits were reported by pharmacies participating in the study for patients with influenza and pharyngitis. According to the results, access to health care improved as patients visited pharmacies outside of opening hours. This prevented them from being admitted to secondary or tertiary care centers as 53.7% did not have access to a primary care provider [12].

In the Diabetes Ten City Challenge (DTCC), a diabetes health management program offered by community pharmacies, the economic and clinical benefits of a control program for patients with diabetes mellitus were evaluated. Through scheduled counseling sessions, 573 patients with diabetes mellitus voluntarily participated in the study. The reduction of medical care costs by 8.5%, as well as the reduction of the average total cost of care per patient, per year, were some of the results that indicated the particularly significant impact of the program on the quality of life of patients and the significant benefits that can be gained from it long-term [13]. A recent study conducted in Malta found that most patients maintain a positive attitude toward pharmacists' expanded responsibilities and the evolution of their roles [13]. Specifically, patients expressed a desire for the development of collaborative care models, which would allow pharmacists to work directly with health professionals (mainly primary care and secondary health care providers), as well as provide diagnostic tests to patients.

The Lancet recently published a study aimed at creating a global health index called the HAQ Index (Health Access and Quality). In this index, 195 countries are rated according to the quality, effectiveness, and accessibility of their health systems. A scoring system was used for 29 diseases and pharmacotherapy adverse effects from 1990-2015. According to the HAQ, Greece ranks 19th for the treatment of diarrhea diseases, diphtheria, whooping cough, measles, epilepsy, appendicitis, upper respiratory diseases, hypertension, and diabetes. Primary healthcare services are provided in an extensive network of pharmacies throughout Greece, and they are easily accessible and affordable [14].

In the present research, participants suggested a wider range of health services. These included systematic vital sign-taking, collaboration with other health scientists such as a physician and nutritionist, and home visits by pharmacists. Pharmacy services are not compensated by insurance agencies in Greece due to the absence of a clear legislative framework regarding their provision. It is purely a private initiative resulting from a lack of capacity in the health system to treat the massive increase in patients. There was no evaluation of the financial benefits of the primary services provided in this study. A literature review found no significant findings regarding pharmacy care. A lot more research is needed to determine the number of resources to be distributed to each service by the relevant health policy bodies.

It will be much easier to understand how pharmacists can play a multifaceted role in the modern era. This is because there are documented studies that demonstrate the importance of pharmacists in health education and prevention, as well as their benefits. A study will also need to investigate the financial benefits associated with the provision of similar health services. This will include the increased flow of patients to public hospitals for seeking primary care. In evaluating the health system, tertiary medical services should not be the only focus. A better quality of services and a higher level of patient satisfaction can be achieved through the cooperation of all health agencies and health professionals.

This study has several limitations. First, the data were collected after the lockdown that had occurred in Athens so the participants were not so willing to fill out the questionnaire. Secondly, the results of our study indicate high patient satisfaction with the services provided by pharmacists during covid pandemic. Nevertheless, it is necessary to be done further research with a larger sample.

## Conclusions

Several significant conclusions were drawn regarding the primary care services provided by pharmacies in this study. Particularly, the pharmacy plays a significant role in health care. Patients-clients trust their pharmacist as a health professional and seek their advice on medication-related issues. Pharmacy is sometimes the primary care station for health services and occupies a dominant position due to its easy access, fast and immediate service, and low cost. There is a good level of general understanding of the pharmacist’s role. As necessary with expanded services increases, it is expected that the demand for further amelioration of primary care roles will also grow. In addition, the collaboration of the pharmacist with other health scientists strengthens the role of the pharmacist in primary care and many cases appear to have a regulatory role in further referring patients where and when they are needed.
